# Die Corona-Pandemie und Multiple Sklerose: Impfungen und deren Implikationen für Patienten – Teil 2: Impfstofftechnologien

**DOI:** 10.1007/s00115-021-01154-5

**Published:** 2021-07-07

**Authors:** Tobias Monschein, Tobias Zrzavy, Micha Löbermann, Alexander Winkelmann, Thomas Berger, Paulus Rommer, Hans-Peter Hartung, Uwe K. Zettl

**Affiliations:** 1 Universitätsklinik für Neurologie, Medizinische Universität Wien, Waehringer Guertel 18–20, 1090 Wien, Österreich; 2 Abteilung für Tropenmedizin und Infektionskrankheiten, Universitätsmedizin Rostock, Rostock, Deutschland; 3 Klinik und Poliklinik für Neurologie, Universitätsmedizin Rostock, Rostock, Deutschland; 4 Klinik für Neurologie, Universitätsklinikum Düsseldorf, Medizinische Fakultät, Heinrich-Heine-Universität, Moorenstraße 5, 40225 Düsseldorf, Deutschland; 5 Klinik und Poliklinik für Neurologie, Neuroimmunologische Sektion, Universitätsmedizin Rostock, Rostock, Deutschland

**Keywords:** Impfstofftechnologie, Genbasiert, Vektor, COVID-19, SARS-CoV-2, Vaccine technologies, Gene-based, Vector, COVID-19, SARS-CoV-2

## Abstract

Im Zusammenhang mit den Herausforderungen durch die weltweit vorherrschende COVID-19-Pandemie kam es zu teils epochalen Fortschritten im Bereich der Impfstofftechnologien. Neben den bereits langjährig eingesetzten Tot‑, Lebend- und proteinbasierten Impfstoffen gewannen im Zuge dieser Gesundheitskrise vektor- und genbasierte Impfstoffe enorm an Bedeutung. Ziel dieser Arbeit ist es daher, einen Überblick über Multiple Sklerose und Impfen, rezente Fortschritte in der SARS-CoV-2-Impfstoff-Landschaft sowie eine detaillierte Auseinandersetzung mit den verschiedenen Impfstofftechnologien zu bieten. Abschließend sollen übersichtsmäßig klare Empfehlungen im Zusammenhang mit krankheitsmodifizierenden Therapien und Impfen bei Multiple Sklerose gegeben werden.

## Abstract (en)

Along with the challenges posed by the globally circulating COVID-19 pandemic, there have been some epochal advances in the field of vaccine technologies. In addition to the traditionally used dead, live and protein-based vaccines, vector-based and gene-based vaccines gained enormous attention in the course of this health crisis. The aim of this article is to provide an overview of multiple sclerosis (MS) and vaccination, recent advances in the SARS-CoV‑2 vaccine landscape as well as a detailed discussion of the various vaccine technologies. Finally, clear recommendations in the context of disease-modifying treatment and vaccination in MS are highlighted.

## Impfen und Multiple Sklerose

Das Impfwesen gehört zu den größten Errungenschaften der Medizingeschichte. Begonnen hat alles in der Medizinhistorie des Abendlandes mit der Inokulation von Kuhpocken durch den englischen Arzt Edward Jenner 1796 [55]. Danach entdeckte Robert Koch erstmals Bakterien als Ursache für den Milzbrand und die Tuberkulose.

In weiterer Folge wurden die ersten Impfstoffe gegen Milzbrand und Tollwut mittels gezüchteter attenuierter Keime durch Louis Pasteur und Emile Roux entwickelt [80]. Diese enormen Fortschritte im Bereich der Vakzinologie setzten sich immer weiter fort, sodass wir inzwischen im Zeitalter der genbasierten Impfstoffe angelangt sind [57]. Die Wirksamkeit von Impfstoffen kann nicht zuletzt durch die Ausrottung der Pocken, beinahe Ausrottung von Poliomyelitis oder der massiven Eindämmung von Erkrankungen wie der Diphtherie, Masern, Mumps oder Röteln zweifellos belegt werden [49]. Das Ziel von Impfprogrammen ist daher nicht nur der Individualschutz, sondern auch die Elimination bzw. das Zurückdrängen von Erkrankungen sowie auch die Verhinderung von Epidemien und das Erlangen einer Herdenimmunität [80].

Impfungen gelten im Allgemeinen als sicher. In diesem Zusammenhang sollte zwischen Impfreaktionen, Impfnebenwirkungen und Impfschäden unterschieden werden [80]. Impfreaktionen sind häufig, harmlos und passagere Beschwerden, welche von Lokalreaktionen über grippeähnliche Symptome bis hin zu „Impfmasern“ im Falle der Masernimpfung reichen können [78]. Im Gegensatz dazu wird eine Impfnebenwirkung durch eine schädliche und unbeabsichtigte Reaktion auf eine Impfung definiert [78]. Der Begriff des Impfschadens leitet sich aus dem Infektionsschutzgesetz ab und ist definiert durch „gesundheitliche und wirtschaftliche Folgen einer über das übliche Ausmaß hinausgehenden gesundheitlichen Schädigung durch eine Schutzimpfung“ [78]. Insgesamt sind Impfnebenwirkungen und -schäden sehr selten [78]. In diesem Zusammenhang ist ein wichtiges Beispiel mit enormen Folgen eine Publikation von Wakefield et al. im Fachjournal *The Lancet* aus dem Jahre 1998 [76]. Hier wurde anhand von nur 12 Kindern und ohne Kontrollgruppe ein Zusammenhang von Autismus und der Masern-Mumps-Röteln-Impfung (MMR) vermutet. Die Studie wurde 2010 zurückgezogen, weil sie falsifiziert wurde, resultierte jedoch in einem extrem relevanten Rückgang der Durchimpfungsraten und entsprechenden Zuwachs der Impfskepsis [80]. Mittlerweile konnte in zahlreichen Aufarbeitungen gezeigt werden, dass es keinen Zusammenhang zwischen der MMR-Impfung und Autismus gibt [70, 71].

Bezugnehmend auf Multiple Sklerose (MS) wurde beispielsweise ein erhöhtes MS-Risiko im Zusammenhang mit der Hepatitis-B-Impfung postuliert [25]. Dies bestätigte sich jedoch ebenfalls in einer großen Fall-Cross-over-Studie von Confavreux nicht [12]. Dem folgend konnte, trotz zahlreicher epidemiologischer Studien, bis heute keine Verursachung oder Auslösung von Multipler Sklerose durch Impfungen nachgewiesen werden, genauso wenig wie das Auslösen eines Schubes (außer in einigen Fallserien zur Gelbfieberimpfung; [15, 35]). Bezüglich der Typhusimpfung wurde sogar ein potenziell positiver Einfluss auf den Erkrankungsverlauf der MS hypothetisiert [86]. Des Weiteren konnte gezeigt werden, dass Impfen mit einer geringeren Wahrscheinlichkeit verbunden ist, innerhalb der nächsten 5 Jahre an MS zu erkranken [23]. Da gerade für MS-Patienten Infektionen ein erhebliches Risiko mit sich bringen, gilt es, gerade diese durch Impfungen zu schützen [16, 42, 86].

## COVID-19-Impfungen und MS

**Figure 1:**
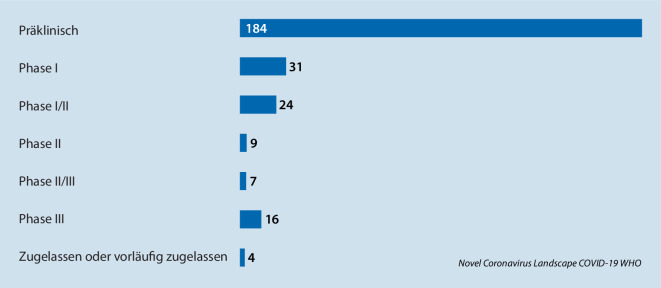

Die insgesamt 275 SARS-Cov-2-Impfstoffe, die sich in der Entwicklung befinden, spiegeln den enormen wissenschaftlichen Fortschritt seit Anbeginn der Pandemie wider (Details siehe Abb. 1**; **[108]).

Weltweit haben es bisher 14 Impfstoffe zur Zulassung geschafft [89]. Davon sind die beiden mRNA-Impfstoffe von BionTech/Pfizer (BNT162b2 am 21.12.2020) und Moderna (mRNA-1273 am 06.01.2021) sowie der nichtreplizierende Vektorvirusimpfstoff von AstraZeneca (ChAdOx1 nCoV-19 am 29.01.2021) und jener von Johnson&Johnson (Janssen COVID-19 Vaccine am 11.03.2021) durch die Europäische Arzneimittelbehörde (EMA) in der Europäischen Union (EU) zugelassen worden [3, 56, 75, 94]. Des Weiteren wurden die nichtreplizierenden Vektorvirusimpfstoffe Sputnik‑V (bereits in Phase II am 11.08.2020) in Russland, Covaxin (BBV152) in Indien (am 02.01.2021 ohne veröffentlichte Wirksamkeitsdaten), „Serum Institute of India – Covishield“ in Indien (gleiche Formulierung wie AstraZeneca) sowie „CanSino: Ad5-nCoV“ in China zugelassen [38, 51, 75, 88]. An Protein-Subunit-Impfstoffen wurden „Anhui Zhifei Longcom – RBD-Dimer“ in China und „FBRI: EpiVacCorona“ in Russland zugelassen [52, 82]. Schließlich wurden 3 inaktivierte Totimpfstoffe („Sinopharm [Beijing] BBIBP-CorV“, „Sinopharm [Wuhan] Inactivated [Vero Cells]“ und „Sinovac CoronaVac“) in China, 1 („Bharat Biotech Covaxin“) in Indien und 1 (Chumakov Center: KoviVac) in Russland zugelassen [81, 84, 87, 89].

Die Wirksamkeitsprofile der mRNA-Impfstoffe sind mit 95 % bei der Prävention von COVID-19 beim BionTech/Pfizer-Impfstoff sowie 94,1 % bei jenem von Moderna als hervorragend anzusehen. Auch das Sicherheitsprofil gilt als sehr gut. Die Nebenwirkungen waren im Wesentlichen auf lokale Reaktionen und milde systemische Reaktionen beschränkt. Beim BionTech/Pfizer-Impfstoff wurden vier schwere Nebenwirkungen (SAEs) berichtet (Schulterverletzung im Zusammenhang mit der Impfstoffverabreichung, axilläre Lymphadenopathie rechts, paroxysmale ventrikuläre Arrhythmie und Parästhesie im rechten Bein; [56]). Im Falle des Moderna-Impfstoffs wurden Fazialisparesen dokumentiert. Diese traten jedoch in beiden Gruppen mit einer Häufigkeit von < 0,1 % auf [3]. Des Weiteren wurden in der Phase-III-Zulassungsstudie des Moderna-Impfstoffs auch Risikogruppen, einschließlich immunsupprimierter Patienten mit HIV-Infektion, eingeschlossen. Daran anschließend wurde für den BionTech/Pfizer-Impfstoff ein Sicherheitsupdate nach der Zulassung veröffentlicht, das keine neuen Sicherheitsaspekte erbrachte [95].

Der Impfstoff von AstraZeneca weist in der gepoolten Analyse gegenüber den mRNA-Impfstoffen mit 70 % eine niedrigere Wirksamkeit auf, wobei die Vergleichbarkeit nur bedingt gegeben ist. Das Sicherheitsprofil war insgesamt ebenfalls gut, dennoch wurden in der klinischen Phase-III-Studie 3 Fälle von transverser Myelitis (TM) dokumentiert [75]. Einem unabhängigen neurologischen Expertengremium zufolge wurden diese als unwahrscheinlich im Zusammenhang mit der Impfung stehend bewertet [75]. Im Detail wurde ein Fall einer bislang unbekannten MS zugeordnet, ein Fall einer in zeitlichen Zusammenhang stehenden Meningokokkenimpfung und der dritte Fall blieb unklar [75]. Insgesamt wurden den Daten der WHO zufolge bereits 899.936.102 Impfdosen (laut WHO bis zum 22.04.2021) verabreicht [90]. An dieser Stelle muss erwähnt werden, dass im Zusammenhang mit dem AstraZeneca-Impfstoff (Vaxzervria) bis zum 04.04.2021 169 Fälle von zerebraler Venenthrombose (CVT) und 53 Fälle von splanchnischer Venenthrombose in der EU und Großbritannien gemeldet wurden (bei über 34 Mio. geimpften Personen; [11, 20, 48, 64, 66, 93]). Bei den bisher dem Paul-Ehrlich-Institut (PEI) gemeldeten Fällen traten diese vorwiegend bei Frauen und in einem Alter < 60 Jahren auf. Des Weiteren kam es in der Regel nach 4 bis 16 Tagen zu den Thrombosen gemeinsam mit einer Thrombozytopenie, hinweisend für eine immunologische Genese [105]. In einer Stellungnahme der Gesellschaft für Thrombose- und Hämostaseforschung (GTH) vom 01.04.2021 wurde hypothetisiert, dass es im Zuge einer impfinduzierten inflammatorischen Reaktion zu einer Immunstimulation mit Antikörperbildung gegen Plättchenantigene kommt. Dies führt dann, wie bei der heparininduzierten Thrombozytopenie (HIT), über den Fc-Rezeptor zu einer massiven Thrombozytenaktivierung und resultiert hier in einer prothrombotischen Thrombozytopenie [99]. Der EMA zufolge überwiegt dennoch weiterhin klar der Nutzen dem Risiko, der STIKO zufolge sollten, basierend auf den genannten Berichten, Menschen unter 60 Jahren nicht mehr mit AstraZeneca geimpft werden und jene mit bereits einer erhaltenen Dosis als 2. Dosis einen mRNA-Impfstoff erhalten [106]. Auch den Johnson&Johnson-Impfstoff betreffend wurden 6 Fälle von CVT gemeldet. Diese stellen jedoch nach einer rezenten Überprüfung durch die FDA (Food and Drug Administration) und das CDC (Centers for Disease Control and Prevention) keinen Grund zur Revidierung der bestehenden Zulassung dar [98].

Im Allgemeinen kann aber von einem sehr guten Sicherheitsprofil der in der EU zugelassenen Impfstoffe ausgegangen werden. Dennoch können theoretisch potenzielle Langzeitnebenwirkungen der neuen genetischen Impfstoffe (Nukleinsäure- und vektorbasierte Impfstoffe) gegenwärtig noch nicht beurteilt werden, weshalb eine entsprechende strenge Observanz nach der Zulassung notwendig ist [13, 46].

Gegen Ende des Jahres 2020 kam jedoch im Zuge der COVID-19-Pandemie ein neues Problem auf, nämlich jenes der diversen Virusmutationen [2]. Die erste detektierte Virusvariante ist der B.1.1.7 Stamm, welcher erstmals in Großbritannien entdeckt wurde [68]. Hier wurde jedoch bis dato von keiner Resistenz gegenüber den impfstoffinduzierten neutralisierenden Antikörpern ausgegangen [18, 47]. Der B.1.351- und P.1-Stamm, die in Südafrika und Brasilien aufkamen, stellen aktuellen Expertenmeinungen zufolge wahrscheinlich kein Problem in Bezug auf die Wirksamkeit der mRNA-Impfstoffe dar [47]. Dennoch erwies sich im Labor-Setting die Variante B.1.351 als teilweise resistent gegen neutralisierende Antikörper, die durch 2 Dosen des „BNT162b2“-Impfstoffs, des „mRNA-1273“-Impfstoffs und des „NVX-CoV2373“-Impfstoffs induziert wurden [18, 68]. Der Impfstoff „ChAdOx1 nCoV-19“ scheint keine protektive Immunantwort gegen den B.1.351-Stamm aufbauen zu können [41]. Ein Problem im Rahmen der Resistenzbildungen ist, dass in der Phase zwischen den ersten und zweiten Impfungen ein suboptimaler Antikörpertiter erreicht wird, welcher zu Resistenzbildungen prädisponiert. Demnach könnte der am 27.02.2021 von der FDA notzugelassene nur einmalig zu verabreichende Impfstoff „Janssen COVID-19 Vaccine“ eine attraktive Alternative/Ergänzung im Zuge der Pandemiebekämpfung darstellen [97]. Erste Real-world-Daten aus Israel zeigen eine erhöhte Inzidenz an Infektionen bei zweimalig geimpften mit der Variante B.1.351 sowie bei einmalig Geimpften mit der Variante B.1.1.7. [30]. Weitere Studien sind notwendig, um die Durchbruchsinfektionen von SARS-CoV‑2 bei Geimpften zu verstehen [21]. Um einen möglichst effektiven und raschen Umgang mit den Virusvarianten zu erreichen, wurde von der EH eine EU-Behörde für die Krisenvorsorge und -reaktion bei gesundheitlichen Notlagen (HERA) ins Leben gerufen. Im Rahmen dessen soll auch das VACCELERATE-Programm bei der Erprobung neuer/angepasster Impfstoffen helfen [96]. Insgesamt ist jedenfalls die möglichst rasche Durchimpfung weltweit essenziell, nicht zuletzt, um die Gefahr weiterer problematischer Virusmutanten zu unterbinden.

## Impfstofftechnologien im Überblick

Durch die Entwicklung und den Einsatz von Impfstoffen zur aktiven Immunisierung wurden viele Infektionskrankheiten deutlich reduziert oder nahezu vollständig verdrängt [49].

Die gegenwärtigen – zum Teil epochalen – Ansätze zur Impfstoffentwicklung beruhen auf unterschiedlichen biotechnologischen Strategien und sind insbesondere im Rahmen der SARS-CoV-2-Pandemie in den Fokus eines großen öffentlichen Interesses gerückt [40, 67].

**Figure 2:**
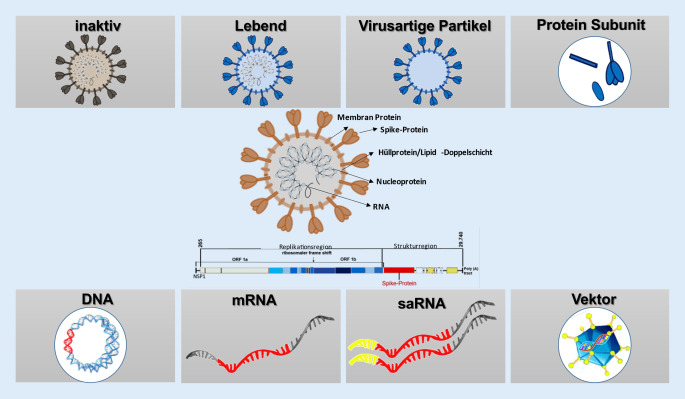

Prinzipiell unterscheiden wir in diesem Indikationsgebiet (Abb. 2):

- inaktivierte Impfstoffe (Totimpfstoffe),
- attenuierte (abgeschwächte) Lebendimpfstoffe,
- proteinbasierte Impfstoffe,
  - Virus-like-particles(VLP)-Impfstoffe,
  - Subunit-(Proteinuntereinheiten‑)Impfstoffe,
- Vektorimpfstoffe,
- Nukleinsäure- (genbasierte) Impfstoffe.

Toxoidimpfstoffe, wie sie zur aktiven Immunisierung gegen Tetanus sowie Diphterie eingesetzt werden, spielen im Kontext der SARS-CoV-2-Pandemie Impfstoffentwicklung keine Rolle und sollen nur aus Vollständigkeitsgründen an dieser Stelle Erwähnung finden.

### Inaktivierte Impfstoffe und abgeschwächte Lebendimpfstoffe

Beide „klassischen“ Impfstoffe verwenden seit langer Zeit gut etablierte Biotechnologien und stellen für viele gegenwärtig in praxi eingesetzte Impfstoffe die Basis dar [55]. Während bei den inaktivierten Impfstoffen das genetische Material des Infektionserregers funktionsunfähig, d. h. nicht mehr replikationsfähig, ist, sind bei den attenuierten Lebendimpfstoffen die Infektionserreger zwar abgeschwächt, aber noch replikationsfähig [10, 32]. Bei beiden Impfstoffen werden nach Applikation eine Reihe von Immunprozessen initiiert, die zur Aktivierung von T‑Helfer-Zellen (CD4^+^-T-Zellen), CD8^+^-T-Zellen und B‑Zellen mit konsekutiver Antikörperproduktion führen und im Endeffekt ein immunologisches Gedächtnis sowohl in T‑ als auch in B‑Lymphozyten (T- und B‑Memory-Zellen) zur Folge haben können [57]. Bei der Impfstoffherstellung und beim klinischen Impfstoffeinsatz sind von Erreger zu Erreger sowohl unterschiedliche „Anzüchtungs-“ und Herstellungsbedingungen als auch ein unterschiedliches Immunisierungspotenzial (Stärke und Langlebigkeit der Immunreaktion) zu bedenken. In der Regel sind Lebendimpfungen immunogener als inaktivierte Impfstoffe, sodass einerseits Zusatzfaktoren (Adjuvanzien – z. B. Aluminiumsalze) in den Impfstoff integriert werden müssen oder andererseits Booster- bzw. Auffrischungsimpfungen notwendig sind, um eine klinisch relevante aktive Immunisierung zu erreichen [59]. Der Nachteil der in der Regel stärker immunogen wirkenden Lebendimpfstoffe ist, dass sie einerseits instabiler sind und andererseits bei Personen mit einem geschwächten Immunsystem gegebenenfalls zur Manifestation der Infektionskrankheit durch die abgeschwächten Impferreger führen können [57].

### Proteinbasierte Impfstoffe

Zu dieser Impfstoffgruppe zählen Virus-like-particles(VLP)-Impfstoffe und Subunit-(Proteinuntereinheiten‑)Impfstoffe. Da viele der als Antigen verwendeten hoch aufgereinigten Proteine meist nicht ausreichend immunogen wirken, müssen diesen Impfstoffen Adjuvanzien (Wirkverstärker) zugesetzt werden. So werden Komponenten des angeborenen „unspezifischen“ Immunsystems in die Nähe der eigentlichen Impfantigene gelockt und führen hier zu einer lokalen Verstärkung der Impfreaktion [57, 59]. Die Influenzavakzine sind typische proteinbasierte (Split‑, Subunit‑)Impfstoffe [77]. Laut WHO sind mindestens drei proteinbasierte Impfstoffe gegen SARS-CoV‑2 in klinischer Prüfung [108].

#### Virus-like-particles-Impfstoffe

Virus-like-particles(VLP)-Impfstoffe sind Pseudovirionen, die aus nichtinfektiösen Partikeln („leere“ Lipidhüllen mit Virusstrukturproteinen als Impfantigen) hergestellt werden, aber genügend virale Proteine enthalten, um eine Immunreaktion auszulösen [57].

#### Subunit-(Proteinuntereinheiten‑)Impfstoffe

Diese Impfstoffe verwenden Teile des Infektionserregers, häufig Proteinfragmente, um die aktive Immunisierung zu initiieren. Der Vorteil ist die Minimierung von Nebenwirkungen, insbesondere des Infektionsrisikos. Nachteile sind, dass die Immunantwort in der Regel schwächer ausfällt, insbesondere, wenn molekulare Strukturen, wie „pathogen-associated molecular patterns“ (PAMPs), dem Impfstoff fehlen.

Aus diesem Grund müssen diesen Impfstoffen häufig Adjuvanzien zur Verstärkung der Immunreaktion beigefügt werden. Da die geimpften Antigene keine Körperzellen infizieren, lösen diese Impfstoffe vorrangig antikörpervermittelte Immunantworten aus [57, 59].

Alle Subunit-Impfstoffe werden mithilfe moderner Biotechnologien unter Verwendung von Bakterien oder Hefen im Rahmen strenger Hygienevorschriften hergestellt. So wird für den rekombinanten Hepatitis-B-Impfstoff der genetische Code für das Impfantigen in Hefezellen integriert, durch diese in großen Mengen produziert und nach Aufreinigung als Impfantigen zur Verfügung gestellt. Durch Zusatz weiterer Komponenten, wie Konservierungsmittel und Adjuvanzien, in diesem Fall Alaun, wird der Impfstoff komplettiert [24, 74].

Auch Polysaccharid- oder Konjugatimpfstoffe werden mithilfe von Bakterien in Bioreaktoren produziert und anschließend durch biotechnologische Verfahren bis zur finalen Impfstoffkomponente aufbereitet [57].

### Vektorimpfstoffe

Bei den Vektorimpfstoffen wird mithilfe viraler Vektoren („Taxi“, Transporter) die genetische Information für das Impfantigen in Körperzellen eingeschleust und wie bei den Nukleinsäureimpfstoffen konsekutiv von diesen gebildet. Im Genaueren führt die intramuskuläre Injektion eines rekombinanten Adenovirusimpfstoffs zu einer Infektion der Muskelzellen, gefolgt von der Expression des Transgens innerhalb von 24 h, mit konkomitanter Auslösung einer angeborenen Immunantwort. Die exprimierten Proteine werden konsekutiv proteasomal abgebaut und anschließend über MHCI CD8^+^-T-Zellen präsentiert oder von professionellen antigenpräsentierenden Zellen (APC) aufgenommen. Schließlich wandern antigenbeladene APC zu den drainierenden Lymphknoten, wo sie in der Lage sind, CD8^+^-, CD4^+^-T-Zellen und B‑Zellen zu aktivieren, und so eine aktive Immunisierung bewirken [14, 29, 39, 62]. Als virale Vektoren stehen beispielsweise das modifizierte Vakziniavirus Ankara (MVA), das gentechnisch hergestellte vesikuläre Stomatitisvirus (rVSV) und insbesondere das Adenovirusserotyp 5 und 26 zur Verfügung.

Das Adenovirus 5 wird z. B. als Vektor für die Impfstoffe von AstraZeneca/University of Oxford (AZD 1222) oder CanSino Biological/Beijing Institute of Biotechnology (Ad5-nCoV) für die zweizeitige Applikation verwendet. Der russische Vektorimpfstoff Sputnik‑V muss ebenfalls zweimal pro Person geimpft werden. Verwendet werden hierbei aber jeweils zwei verschiedene Vektoren (Adenovirusserotyp 5 und 26), um die Immunogenität gegen den Vektor zu minimieren [37, 62, 75, 85].

Prinzipiell unterscheidet man replizierende und nichtreplizierende Vektorimpfstoffe. Nichtreplizierende Vektorimpfstoffe können keine neuen Viruspartikel (virale Vektoren) herstellen. Sie produzieren nur das Impfantigen. Im Gegensatz hierzu können replizierende Vektorimpfstoffe neue Viruspartikel in primär infizierten Körperzellen bilden, die nachfolgend weitere Zellen befallen und gemeinsam das Impfantigen bilden. Durch die hierbei induzierte Immunreaktion kommt es nachfolgend zur Limitierung der Viruspartikelreplikation [62]. Der erste in der EU zugelassene Vektorimpfstoff (November 2019) war der Ebola-Impfstoff Ervebo (rVSV-ZEBOV; [22]). Bisher sind ausschließlich nichtreplizierende Vektorimpfstoffe im klinischen Einsatz zur aktiven Immunisierung von Infektionserregern.

Klassisch werden virale Vektoren in Zellen gezüchtet, die an ein Substrat gebunden sind und nicht in freischwebenden Zellen. Dies ist sowohl für die Großproduktion als auch für die Skalierbarkeit von Nachteil. Aus diesem Grund geht die Forschung und Entwicklung in Richtung von Suspensionszelllinien, mit denen virale Vektoren in Bioreaktoren gezüchtet werden können. Der Zusammenbau der Vektorimpfstoffe ist ein komplexer Prozess mit Kontaminationsrisiken, die umfangreiche Sicherheitstests erforderlich machen [19, 58, 61].

Eine besondere klinische Herausforderung der Vektorimpfstoffe stellt eine bereits im Vorfeld der Impfung erworben Immunantwort gegen den viralen Vektor dar, die zu einer verringerten aktiven Immunisierung (Impfantwort) gegen das Impfantigen führen kann [17]. Diese potenzielle Antivektorimmunität macht es zudem problematisch, eine notwendige Booster-Immunisierung (zweite oder mehrfache Impfung) mit dem gleichen Vektor durchzuführen. Dies könnte ein möglicher Grund für die prozentual niedrigere Wirksamkeit des AstraZeneca-Impfstoffs sein. Die präexistierende oder durch die aktive Erstimmunisierung induzierte spezifische Immunantwort gegen den viralen Impfvektor stellt jedenfalls eine besondere Herausforderung im klinischen Großeinsatz dar und ist aktuell noch nicht abschließend zu bewerten.

### Genbasierte Impfstoffe

Genbasierte Impfstoffe, auch Nukleinsäureimpfstoffe genannt, verwenden entweder mRNA (Messenger-Ribonukleinsäure) oder DNA (Desoxyribonukleinsäure), um Körperzellen die Anleitung zur Produktion des Impfantigens zu geben und nachfolgend die aktive Immunisierung zu initiieren [26, 69]. Das heißt, der Körper der geimpften Person stellt, wie bei den Vektorimpfstoffen, selbst das Impfantigen her [57]. Bei DNA-Impfstoffen wird die das Antigen kodierende DNA chemisch synthetisiert und mithilfe spezifischer Enzyme in ein Bakterienplasmid inseriert. Das rasante Kopieren dieses Plasmids erfolgt initial in sich schnell teilenden Bakterien, mit nachfolgender Plasmidisolierung und -aufreinigung [33, 83]. Bei mRNA-Impfstoffen wird in der Regel die genetische Information (mRNA) für das Impfantigen (Protein) in das Zytosol von Körperzellen eingeschleust [54].

Um den Eintritt ins Zytosol zu erleichtern, kann die relativ instabile mRNA in Liposomen oder Lipidnanopartikel (LNP) verpackt werden. Im Zytosol wird die mRNA an Ribosomen gebunden und die Bildung eines Polypeptids (Impfantigen) katalysiert. Das Impfantigen wird von den produzierenden Körperzellen an der Zelloberfläche dargeboten und/oder von diesen Körperzellen liberiert. Über beide Wege wird das Impfantigen von Immunzellen erkannt. Ziel der immunologischen Prozessierung ist die Generierung von T‑ und B‑Gedächtnislymphozyten („memory cells“; [43, 54]). Eine Modifikation von mRNA-Impfstoffen besteht darin, dass als genetischer Bauplan für das Impfantigen selbstreplizierende oder selbstamplifizierende RNA (saRNA) verwendet wird. Dies ist beispielsweise bei dem Impfstoff „BNT 162c2“ der Fall (im Gegensatz zu „BNT162b2“ noch nicht zugelassen; [8, 60]). saRNA-Impfstoffe stammen vom alphaviralen Genom mit zwei offenen Leserahmen („open reading frame“ [ORF]; [9]). Der erste ORF codiert für die RNA-abhängige RNA-Polymerase (Replikase) und der zweite ORF für das Strukturprotein, in diesem Fall das Impfantigen, z. B. das Spike-Protein des SARS-CoV‑2.

Der Vorteil dieser nukleinsäurebasierten Technologie ist die relativ einfache und schnelle Herstellung der genbasierten Impfstoffe, sobald die Nukleinsäuresequenz des Impfantigens bekannt ist. Dies ist insbesondere von großer praktischer Relevanz, wenn neu auftretende epidemisch, pandemisch oder schnell mutierende Infektionserreger eine Rolle spielen [54]. Nachteile der mRNA-Impfstoffe sind einerseits logistische Probleme (Lagerung und Transport bei −20 bis unter −70 °C) und andererseits die sehr begrenzten praktischen Erfahrungen bzw. fehlenden Langzeitergebnisse mit diesem Impfstofftyp im Rahmen von Infektionserkrankungen [54].

Im Rahmen der SARS-CoV-2-Pandemie sind in der EU bisher 2 humane mRNA-Impfstoffe (von BioNTech/Pfizer [BNT 162b2; Tozinameran, Comirnaty] am 21.12.2020 und von Moderna Biotech [mRNA-1273; Moderna COVID-19 Vaccine] am 06.01.2021) zugelassen worden [3, 56]. Bisher gibt es keine Zulassung für einen humanen DNA-Impfstoff gegen eine Infektionserkrankung.

## Immuntherapien (DMTs) in der MS und Impfen

Allgemein sollten stets vor der Initiierung einer DMT der aktuelle Immunstatus genauso wie mögliche Kontraindikationen, insbesondere Lebendimpfstoffe betreffend, berücksichtigt werden [34, 36]. Für Totimpfstoffe wird ein Mindestabstand von 2 Wochen und für Lebendimpfstoffe von 4 Wochen vor Initiierung einer Immuntherapie empfohlen. Um die Wahrscheinlichkeit einer möglichst potenten Immunantwort zu erhöhen sind jedoch unabhängig vom Impfstoff 6 Wochen zu bevorzugen, sofern dies die individuelle Situation erlaubt [63, 104]. Gerade Menschen mit Multipler Sklerose werden alle Impfungen entsprechend den regionalen Empfehlungen empfohlen, um so die Infektionswahrscheinlichkeit und die damit verbundenen Risiken zu reduzieren [16, 31, 107]. Eine Ausnahme stellt die Gelbfieberimpfung dar, da hier die Auslösung eines Schubes durch die Impfung nicht ausgeschlossen werden kann [15]. Eine Absprache mit dem behandelnden Neurologen sowie entsprechende Risiko-Nutzen-Evaluation werden empfohlen.

**Figure 3:**
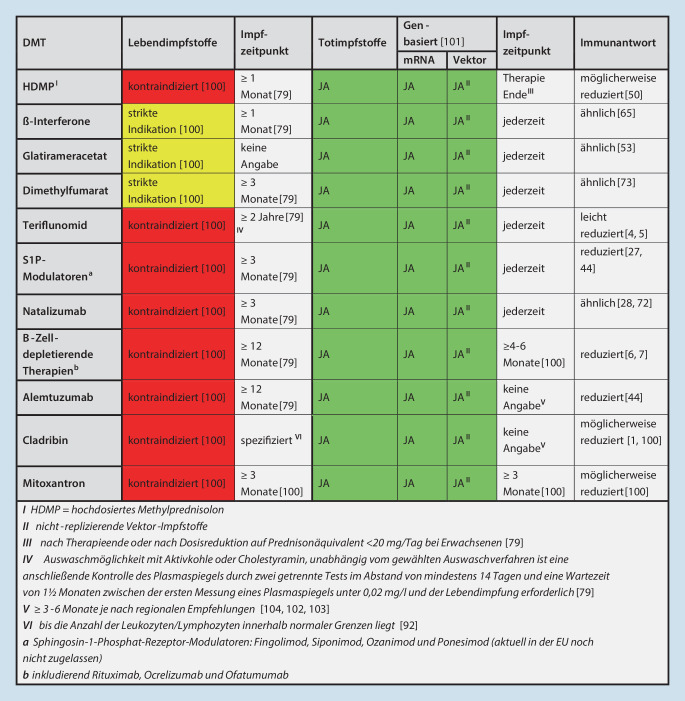

Einen Überblick über die Empfehlungen für Lebend‑/Tot- und genbasierte Impfstoffe in Abhängigkeit von der jeweiligen DMT gibt Abb. 3. Des Weiteren ist es wichtig, die entsprechenden Zeitintervalle einzuhalten, um bei Lebendimpfstoffen eine potenzielle Erkrankung zu verhindern und bei andersartigen Impfstoffen eine protektive Immunantwort aufbauen zu können. Insbesondere bei B‑Zell-depletierenden Therapien ist bei T‑Zell-unabhängigen Antigenen von einer reduzierten Immunantwort auszugehen (VELOCE-Studie; [6]).

Bezugnehmend auf die SARS-CoV-2-Impfstoffe gilt es zu erwähnen, dass in sämtlichen Zulassungsstudien Patienten mit Autoimmunerkrankungen unter Immuntherapie nicht berücksichtigt wurden und dies somit einen klaren Limitierungsfaktor darstellt.

## Schlussfolgerungen

Im Zuge der rezenten COVID-19-Pandemie wurden bisher 2 mRNA-Impfstoffe sowie 2 nichtreplizierende Vektorvirusimpfstoffe von der Europäischen Arzneimittelbehörde (EMA) zugelassen [3, 56, 75, 94]. Diese zeigten allesamt eine sehr gute Wirksamkeit sowie auch ein sehr gutes Sicherheitsprofil. Somit kann anschließend an die Empfehlungen der Internationalen Multiple Sklerose Vereinigung (MSIF) eine klare Empfehlung zur Impfung für alle MS-Patienten, immer in Rücksprache mit dem behandelnden Neurologen, ausgesprochen werden [101]. Die rasche Durchimpfung der Bevölkerung, insbesondere Menschen mit MS, zählt zu den wichtigsten Maßnahmen im Kampf gegen die dominierende COVID-19-Pandemie (Tab. 1).

**Table 1:** 

Land – Fachgesellschaft	Weiterführende Links
Österreich – ÖGN, ÖMSG	https://www.oegn.at/covid-19/covid19-und-multiple-sklerose-ms/ https://www.oemsg.at/news/
Deutschland – DGN, KKNMS	https://dgn.org/ https://www.kompetenznetz-multiplesklerose.de/
Schweiz – SNG, SMSG	https://www.swissneuro.ch/untitled87 https://www.multiplesklerose.ch/de/aktuelles/detail/informationssammlung-zu-ms-corona/
